# Flexible sensor patch for continuous carbon dioxide monitoring

**DOI:** 10.3389/fchem.2022.983523

**Published:** 2022-09-27

**Authors:** Zach Hetzler, Yan Wang, Danny Krafft, Sina Jamalzadegan, Laurie Overton, Michael W. Kudenov, Frances S. Ligler, Qingshan Wei

**Affiliations:** ^1^ Department of Chemical and Biomolecular Engineering, NC State University, Raleigh, NC, United States; ^2^ Department of Electrical and Computer Engineering, NC State University, Raleigh, NC, United States; ^3^ Biomanufacturing Training and Education Center (BTEC), NC State University, Raleigh, NC, United States; ^4^ Department of Biomedical Engineering, Texas A&M University, College Station, TX, United States

**Keywords:** CO_2_, in-line sensor, flexible sensor, process analytical technology, cell culture, biomanufacturing

## Abstract

Monitoring and measurement of carbon dioxide (CO_2_) is critical for many fields. The gold standard CO_2_ sensor, the Severinghaus electrode, has remained unchanged for decades. In recent years, many other CO_2_ sensor formats, such as detection based upon pH-sensitive dyes, have been demonstrated, opening the door for relatively simple optical detection schemes. However, a majority of these optochemical sensors require complex sensor preparation steps and are difficult to control and repeatably execute. Here, we report a facile CO_2_ sensor generation method that suffers from none of the typical fabrication issues. The method described here utilizes polydimethylsiloxane (PDMS) as the flexible sensor matrix and 1-hydroxypyrene-3,6,8-trisulfonate (HPTS), a pH-sensitive dye, as the sensing material. HPTS, a base (NaOH), and glycerol are loaded as dense droplets into a thin PDMS layer which is subsequently cured around the droplet. The fabrication process does not require prior knowledge in chemistry or device fabrication and can be completed as quickly as PDMS cures (∼2 h). We demonstrate the application of this thin-patch sensor for in-line CO_2_ quantification in cell culture media. To this end, we optimized the sensing composition and quantified CO_2_ in the range of 0–20 kPa. A standard curve was generated with high fidelity (*R* ^2^ = 0.998) along with an analytical resolution of 0.5 kPa (3.7 mm Hg). Additionally, the sensor is fully autoclavable for applications requiring sterility and has a long working lifetime. This flexible, simple-to-manufacture sensor has a myriad of potential applications and represents a new, straightforward means for optical carbon dioxide measurement.

## Graphical Abstract

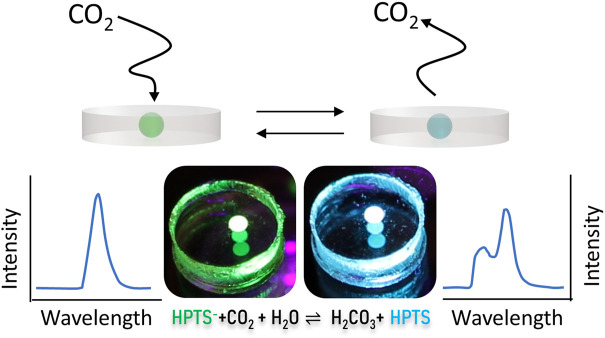

## Introduction

Carbon dioxide (CO_2_) monitoring sees application in a broad range of fields, including waste water treatment (Park and Craggs, 2010), environmental monitoring (Wolfbeis et al., 1998), food science (Neethirajan et al., 2009), and biopharmaceutical production (Ge et al., 2005). In biomanufacturing, cell culture is a cornerstone upstream method for the generation of a wide variety of therapeutic products (Bielser et al., 2018; Bort et al., 2012). CO_2_ is one of the key metabolites used to assess cell population health and as such is regarded as a critical process parameter (CPP) with direct impacts, not only on cell growth, but on final product attributes. In an ideal cell culture system, an in-line CO_2_ sensor would generate real-time data to be automatically fed into process control loops. Widespread CO_2_ sensor integration has not yet been achieved and is thus an unmet critical goal of process analytical technology, a framework for achieving ideal process control as described by the FDA (Food and Drug Administration, 2004).

Currently, the most widely used CO_2_ sensor format is the Severinghaus electrode (Severinghaus and Bradley, 1958). It has now been frequently used in blood-gas analyzers, devices critical to the medical field. The design of the Severinghaus electrode has remained relatively unmodified since its conception in the late 1950s. The Severinghaus principle relies upon a pH probe immersed in a bicarbonate electrolyte solution housed within a CO_2_ permeable membrane. When CO_2_ interacts with the bicarbonate solution, the pH is lowered by the formation of carbonic acid, and the pH change is then correlated to a CO_2_ concentration. Probes relying on the Severinghaus principle have been used in the biopharmaceutical industry as in-line sensors for real-time detection and have become a gold analytics standard. However, the cost and maintenance associated with this detection format are often left unaddressed. In addition, Severinghaus electrodes typically require regular replacement of the reference electrolyte solution as well as replacement of the gas-permeable membrane (Mills, 2009). These high upfront and ongoing maintenance costs can make a Severinghaus electrode prohibitively expensive.

More recently developed CO_2_ sensors rely upon optical detection due to their more straightforward design and use (Wolfbeis et al., 1998; Ge et al., 2005; Mills and Chang, 1994a; Malins and Maccraith, 1998; Chu and Lo, 2008; Dansby-Sparks et al., 2010; Chatterjee and Ayusman, 2015). To couple CO_2_ monitoring with optical detection, a sensing material with spectral behavior in the visible range that changes in the presence of CO_2_ must be chosen. Numerous pH-sensitive, colorimetric, and fluorescent dyes have been incorporated into optochemical CO_2_ sensors. Many optical sensors utilizing 1-hydroxypyrene-3,6,8-trisulfonate (HPTS) have been demonstrated. Dissolved CO_2_ in water forms carbonic acid, which dissociates into bicarbonate and carbonate ions. Thus, HPTS must be in an aqueous environment to detect the acidification of water from dissolved CO_2_. To date, most sensors prepared with HPTS involve complicated ion-pairing loading techniques (Wolfbeis et al., 1998; Mills and Chang, 1993), or sol-gel fabrication (Malins and Maccraith, 1998; Dansby-sparks et al., 2010) to immobilize HPTS in an aqueous environment within a hydrophobic matrix. Such sensors are thus difficult to fabricate, requiring complex chemistry steps, limiting their use to research labs where intricate fabrication processes are tolerable. Widespread industrial adoption of a new CO_2_ sensor format will require a fabrication process that is repeatable, simple, and easily scaled up.

To this end, we have developed a very simple approach to CO_2_ sensor fabrication that can be done in ∼2 h that meets these design requirements. This fast, simple sensor fabrication method requires no chemical reactions. A mixture of HPTS, base (NaOH), and glycerol are loaded in a thin polydimethylsiloxane (PDMS) membrane, which is subsequently cured. The hydrophobic PDMS matrix also serves as the sensing materials’ protective layer and is highly permeable to a broad range of small gas molecules (e.g., CO_2_, O_2_, H_2_, etc.) (Rao et al., 2007). This PDMS-based CO_2_ sensor is capable of real-time optical gas monitoring due to the high gas permeability of PDMS and high optical transmittance. While there are many potential applications for our CO_2_ sensor, we demonstrated its utility for in-line continuous monitoring of CO_2_ in cell culture media in a commercially relevant bioreactor.

## Materials and methods

### Sensor fabrication

The CO_2_ sensor utilizes a pH-sensitive dye, 8-hydroxypyrene-1,3,6-trisulfonate (HPTS) entrapped in a thin hydrophobic PDMS membrane. This high-speed fabrication method uses only a few reagents: PDMS (Sylgard 184, Dow-Corning), HPTS (Fisher Scientific), sodium hydroxide (Fisher Scientific), and glycerol (Fisher Scientific). PDMS was produced by mixing the base and curing agents at a 10:1 (w/w) ratio, then degassed in a vacuum chamber to remove bubbles introduced during mixing. 3D-printed wells were glued to a glass slide to create the CO_2_ sensor patch fabrication mold. PDMS was poured into the glass slide mold and then cured on a 95°C hot plate for 30 min to generate a base layer (<1 mm thick). The slide was removed from the hotplate, and approximately 1 mm of new PDMS solution was poured onto the cured PDMS layer. HPTS, NaOH, and glycerol were combined to create a CO_2_ sensing solution comprised of 50% glycerol (v/v), 1.9 mM HPTS, and 50 mM NaOH. 0.75 uL of the sensing solution was deposited onto the top uncured layer of PDMS. The high density of the sensing droplet due to glycerol induces gravity-based deposition of the sensing droplet (< 1 mm diameter) into the uncured PDMS layer to the surface of the cured layer (Supplementary Video S1). After sensing droplet deposition, the PDMS was cured on a hot plate before cutting the circular sensor with a standard hole puncher, generating a 6 mm diameter flexible sensor.

### Benchtop optical set-up

All preliminary sensor development and optimization was conducted with a benchtop optical detection system we constructed. Excitation light was supplied by a mounted 1,000 mW 405 nm LED (M405L4, Thorlabs) and passed through a 405 nm bandpass filter (FB400-10, Thorlabs). Excitation/Emission light was transmitted to and from the PDMS sensor via fiber optic cable (FP200URT, Thorlabs). Emission light was filtered with a long pass 425 nm filter (84-742, Edmund Optics) before signal digitization via flame miniature spectrometer (Ocean Optics). A simplified schematic of the optical detection system can be seen in Supplementary Figure S1. Data acquisition was performed via OceanView software with 1 s integration time.

### In-line bioreactor CO_2_ quantification

CO_2_ concentration was quantitatively measured in real-time in an in-line sensor format in a cell culture bioreactor to demonstrate accurate, robust, and sensitive analytical metrics. To this end, 2 L glass benchtop bioreactors (Sartorius Stedim) with gas flow through a microsparger, agitation, and temperature controlled by a BIOSTAT B Plus tower (Sartorius Stedim) were used to create cell-culture conditions in the water. Percent CO_2_ control was managed by sparging CO_2_, balanced with N2, via flow control with rotameters (Supplementary Figure S3B). Confirmation of %CO_2_ in the media was confirmed offline with a Beckman Coulter Vi-CELL MetaFLEX bioanalyte analyzer. The temperature was maintained at 37°C, and the agitation rate was held at 200 rpm.

## Results and discussion

### Sensor fabrication and readout

**FIGURE 1:**
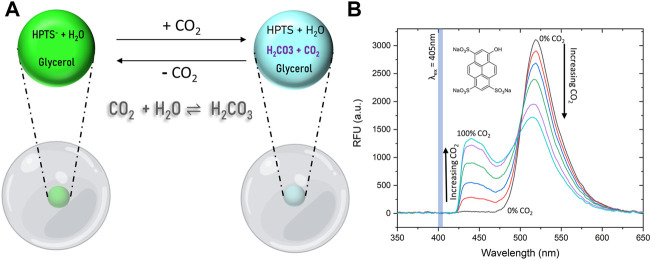
Sensing Mechanism and spectral behavior of HPTS dye. **(A)** HPTS exhibits a strong emission peak at 515 and 455 nm in its deprotonated and protonated forms, respectively. Exposure of the dye in an aqueous media to CO_2_ lowers the pH and HPTS is increasingly protonated with increasing percent CO_2_ levels. **(B)** Fluorescence emission spectra of HPTS entrapped in PDMS upon exposure to increasing CO_2_ concentrations show as HPTS is protonated by an increasingly acidic environment, the deprotonated dye peak decreases and the protonated dye peak increases.

We utilized the pH-sensitive fluorophore, 8-hydroxypyrene-1,3,6-trisulfonate (HPTS), to construct the CO_2_ sensor due to its well-known pH-dependent fluorescent property (Figure 1). The deprotonated form of HPTS fluoresces at about 515 nm, and the protonated form of the dye fluoresces at about 455 nm (Figure 1B) (Chu and Lo, 2009). For use as a CO_2_ sensor, HPTS must be in an aqueous environment. Upon introduction of CO_2_ into an aqueous environment, an equilibrium with carbonic acid is formed as in the reactions below (Mills and Chang, 1994b):

$$CO_{2}\left(g\right)\overset{k_{H}}{⇋}CO_{2}\left(aq\right)$$ (1)

$$CO_{2}\left(aq\right)+H_{2}O\overset{k_{1}}{⇋}H^{+}+HCO_{3}^{-}$$ (2)

$$HCO_{3}^{-}\overset{k_{2}}{⇋}H^{+}+CO_{3}^{2-}$$ (3)

$$D^{-}+H^{+}\overset{k}{⇋}DH$$ (4)

$$\%CO_{2}\left(g\right)=100\left(\frac{{\left[H^{+}\right]}^{3}+{\left[H^{+}\right]}^{2}\left[Na^{+}\right]-k_{w}\left[H^{+}\right]}{k_{H}k_{1}\left(\left[H^{+}\right]+2k_{2}\right)}\right)$$ (5)

where $k_{H}$ is the reciprocal of Henry’s law constant; $k_{1}$ and $k_{2}$ are the 1st and 2nd dissociation constants for carbonic acid, respectively, and k is the reciprocal of the dissociation constant of the pH indicating dye. Gaseous CO_2_ will first dissolve into the aqueous phase, following Henry’s law (Equation 1). CO_2_ in the aqueous phase then reacts with water to form carbonic acid (H_2_CO_3_), a relatively unstable molecule that quickly dissociates to form bicarbonate (HCO_3_^−^) and carbonate ion (CO_3_^2-^) (Equation 2 and Eq. 3). The dissociated hydrogen ion (H^+^) will be captured by the deprotonated pH indicating dye (D^−^), and converted into the protonated form (DH), resulting in fluorescence emission peak shift and intensity change (Equation 4 and Figure 1B). Reactions 1 through 3 can be combined along with the electro-neutrality equation to yield an analytical relationship for %CO_2_ (g) in the gas phase and [H^+^] in the solution (Eq. 5). This relationship was first derived by Severinghaus (Severinghaus and Bradley, 1958) and has been leveraged by numerous others for %CO_2_ determination and sensor optimization (Mills and Chang, 1994b).

**FIGURE 2:**
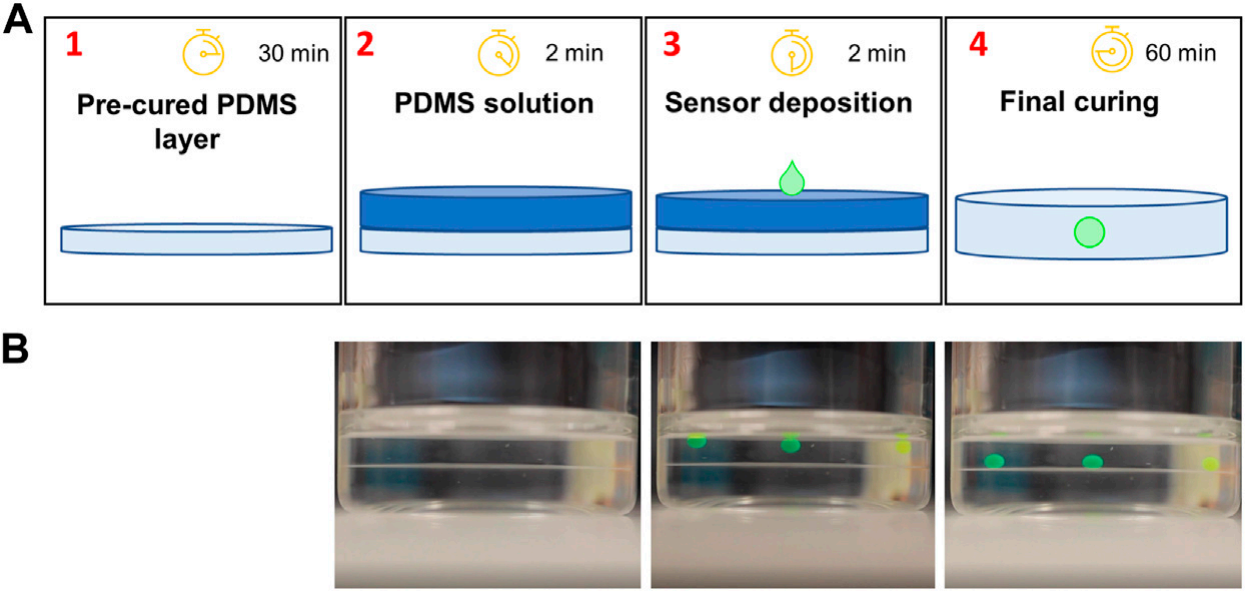
CO_2_ sensor fabrication. **(A)** A thin basal membrane of PDMS is deposited and pre-cured in 30 min or less (1); then a second layer of PDMS is deposited onto the first (2); next, the sensing solution comprised of HPTS dye, NaOH, and glycerol is deposited onto uncured PDMS (3), causing it to sink into the uncured polymer matrix, which is finally cured (4). **(B)** Representative video frames from Supplementary Video S1 showing the deposition of the sensing droplets over time. The interface between cured and uncured PDMS can be observed, notably in the final frame once the sensor droplets reach the cured layer.

**FIGURE 3:**
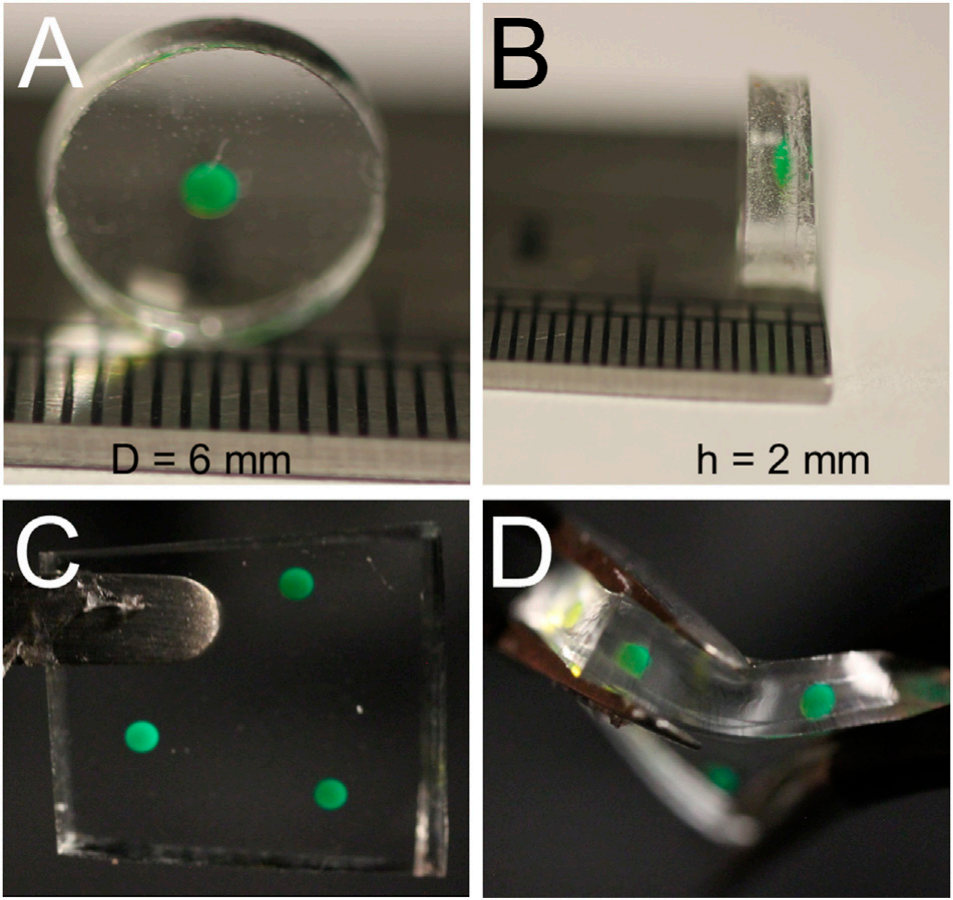
Physical Attributes of Fabricated Sensor. Our sensor was cut from a larger PDMS membrane using a 6 mm punch **(A)**, and the mimimum thickness we achieved was slightly less than 2 mm **(B)**. Using a smaller volume of HPTS allowed for thinner membranes, but reliably aligning the fiber optic sensor with a CO_2_ sensing droplet with a volume under 0.75 uL became the limiting factor **(C)** Multiple sensor compositions can easily be deposited in any 2D configuration allowing for rapid multiplex development. **(D)** Sensors made from PDMS have the added benefit of a high level of stretchability and flexibility, allowing for deployment in diverse geometric configurations.

The potential applications of this CO_2_ sensor are broad; however, we demonstrated the power and flexibility of the sensor for in-line continuous CO_2_ monitoring in a cell culture bioreactor. To this end, essential design considerations included optical transparency, hydrophobicity, autoclavability, and gas permeability. To meet these needs, polydimethylsiloxane (PDMS) was chosen as the matrix in which the pH-sensitive HPTS would reside. Fabrication of the PDMS-based sensors was a simple four-step process (Figure 2) that was adopted from the literature (Faccio et al., 2019), which involves no complex chemical reactions. In brief, a small aqueous volume containing HPTS, water, sodium hydroxide, and glycerol was pipetted onto a thin layer of uncured PDMS solution on top of a pre-cured PDMS substrate (Figure 2A). Glycerol raises the density of the droplet beyond that of PDMS, such that the droplet sinks into the uncured PDMS due to gravity-based deposition. The whole patch is then fully cured, resulting in a hydrophobic, gas permeable sensor responsive to CO_2_; the sensor can be deployed in a variety of environments for which other optochemical CO_2_ sensors may be ill-suited. The whole fabrication process can be done within 120 min (Figure 2B, and Supplementary Video S1). The prepared sensor patch can be constructed in different shapes, dimensions, and thicknesses < 2 mm (Figure 3). The sensor patch is optically transparent, amenable to the addition of multiple dyes for multiplexed responses (Figure 3C), and highly deformable for integration onto nonplanar surfaces (Figure 3D)—qualities very different from most existing CO_2_ sensors.

The responsiveness of the CO_2_ sensor patch was measured using a simple benchtop optical detection system. A schematic of the set-up can be seen in Supplementary Figure S1A (for gas-phase CO_2_) and Supplementary Figure S2A (for dissolved CO_2_). Once the responsiveness and mechanism of CO_2_ detection were established in the simplified gas-phase format, we tested for CO_2_ in the liquid phase using a mass-flow controller to control CO_2_ concentrations (Supplementary Figure S2A), followed by testing in a real 2 L bioreactor (Supplementary Figure S3). HPTS absorbs light around 400 and 460 nm; the protonated form of the dye fluoresces at 455 nm while the deprotonated form of the dye fluoresces at 515 nm) (Chu and Lo, 2008). An optical set-up with an excitation wavelength of 405 nm was chosen to monitor the protonated form of the dye most accurately and allow for future multiplexed monitoring with other dyes with similar absorption spectra. As a consequence of choosing an excitation wavelength (405 nm) corresponding to the protonated form of the dye, we found that spectral behavior of the deprotonated dye, corresponding to an emission wavelength centered around 515 nm, was not a suitable region to analyze CO_2_ concentration. Fluorescence emission at 515 nm was occasionally erratic and overall less sensitive to changes in CO_2_ concentration when compared to the peak centered around 455 nm (Supplementary Figure S1B, Supplementary Figure S5). For the proof-of-concept dissolved CO_2_ measurement, a gas-flow system sparging CO_2_ and N_2_ in a glass beaker was used to mimic the bioreactor (Supplementary Figure S2A). The dissolved CO_2_ and pH were monitored using the sensor patch and a commercial pH probe, respectively. The fluorescence signal of the CO_2_ sensor patch and pH value at different CO_2_ concentrations were inversely proportional (Supplementary Figure S2B), verifying the sensing mechanism.

### Sensor optimization

Similar to the Severinghaus electrode, where the addition of bicarbonate increases the sensitivity of the pH-based sensor, various concentrations of sodium hydroxide in the PDMS-encapsulated dye droplet were investigated to determine optimal sensor chemistry. Several sensor patches were fabricated with final concentrations of 0.005, 0.015, 0.02, 0.025, 0.05, and 0.1 M sodium hydroxide, identical concentrations of HPTS dye (1.9 mM), and glycerol. Each sensor was then installed in a 2 L bioreactor inside a probe assembly, and real-time measurements were taken in water with atmospheric concentrations of CO_2_ as well with 20 kPa partial pressure (∼20%) CO_2_. In most cell culture scenarios, process control demands CO_2_ is maintained in a range under 20 kPa (Blombach and Takors, 2015), these operating conditions informed our targeted maximum working range for the sensor.

**FIGURE 4:**
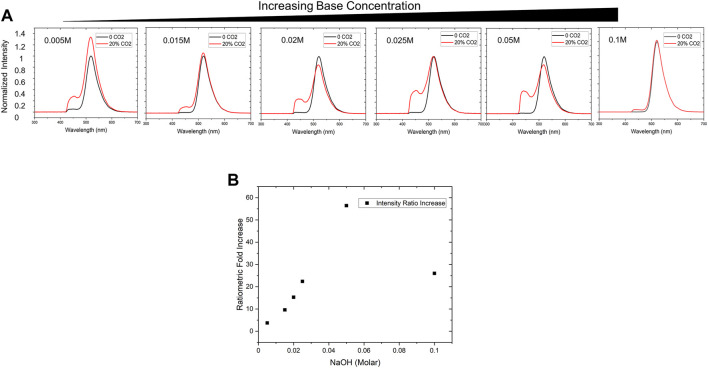
Sensor Chemistry Optimization **(A)** Base concentration is an important factor to optimize sensitivity for the working range of interest. We empirically tested NaOH concentrations ranging from 0.005 to 0.1 M via in-line testing in a 2 L bioreactor and monitored the signal between 0—20% CO_2_ to evaluate maximum spectral shift. **(B)** By evaluating ratiometric increase (final/initial ratiometric signal intensities), the optimal condition of 0.05 M NaOH provided the most significant change over the desired working range.

In our tests, increasing sodium hydroxide concentration from 0.005 to 0.05 M increased the protonated HPTS peak intensity ( $\lambda_{\mathrm{em}}=455\mathrm{nm}$ ) (Figure 4). Interestingly, peak emission intensity for the 0.05 and 0.005 M NaOH conditions were 441 and 455 nm, respectively, suggesting a higher concentration of sodium hydroxide shifts the protonated HPTS peak toward higher energy wavelengths (Figure 4A). We found that sodium hydroxide concentrations at and below 0.01 M NaOH did not maintain a stable spectral baseline after fabrication (Supplementary Figure S4). At lower base concentrations, a peak indicative of the protonated form of the dye would appear after 24 h, suggesting the sensor was influenced by the CO_2_ in the atmosphere. Each sensor’s sensitivity was also assessed through ratiometric intensity change (Figure 4B). The ratiometric intensity is defined as the ratio of the 455 nm peak area (protonated) relative to the 515 nm peak area (deprotonated) (Eq. 6). Utilizing this analysis methodology helps account for erroneous overall intensity increases that occasionally occur during testing. This erroneous emission intensity increase is most apparent in overall spectrum for the 0.005 M NaOH condition (Figure 4A). By normalizing the integrated peak area for the protonated dye against the peak area for the deprotonated dye before and after exposure to 20% CO_2_, any erroneous intensity change is mitigated.

$$\mathrm{Ratiometric}\;\mathrm{Fold}\;\mathrm{Increase}={\left[I_{455}/I_{515}\right]}_{\mathrm{final}}/{\left[I_{455}/I_{515}\right]}_{\mathrm{initial}}$$ (6)

Several factors can be attributed to the presence of a small peak prior to exposure to 20% CO_2_. A likely explanation could be due to acidification of the dye droplet by atmospheric levels of CO_2_ as a result of very low sodium hydroxide concentration. The data suggested that sensor sensitivity increased with sodium hydroxide concentration up to an optimal level at around 0.05 M (Figure 4B).

**FIGURE 5:**
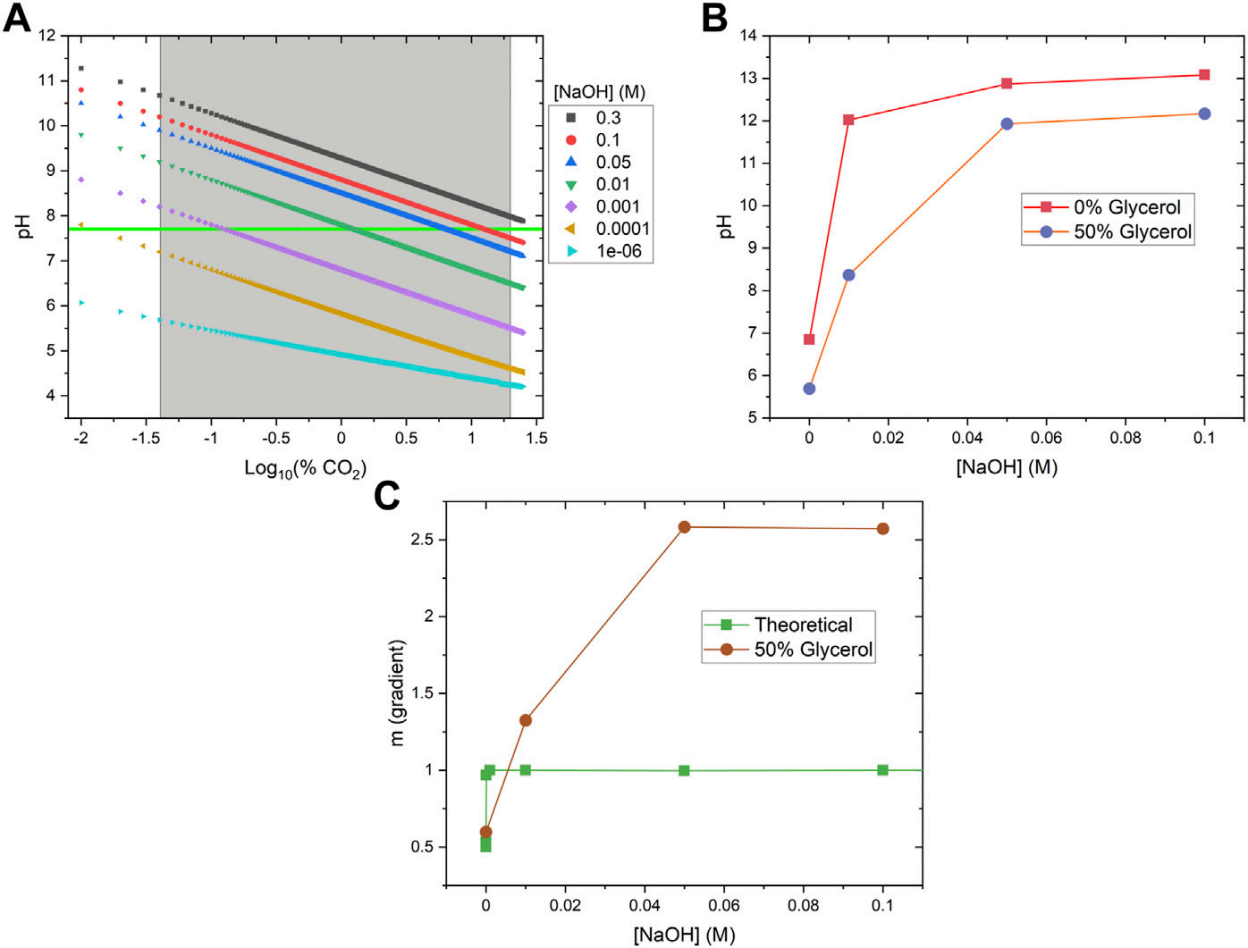
Theoretical and Experimental Validation of Sensor Working Principle. **(A)** pH values of various NaOH solutions were theoretically calculated across the %CO_2_ working range (gray box). The pKa of HPTS is 7.7, and can be seen as the horizontal green line. **(B)** Solutions containing 50% glycerol have significantly lower pH than the corresponding solutions without glycerol. **(C)** The theoretical (green squares) and measured (red circles) sensitivity (or pH gradient change, m) under different base conditions. In each case, above a certain base concentration, change in pH reached a maximum. Solutions containing 50% glycerol reached a maximum at a higher base concentration, resulting from reduced pH values.

A theoretical model was also developed to better understand the influence of base concentration on the sensor performance. Using Equation 5, we numerically calculated the pH of aqueous solutions when exposed to different levels of CO_2_ (from atmospheric level to 20 kPa (20%)) in the presence of different NaOH concentrations (ranging from 1e-6 to 0.3 M) (Figure 5A). From this data, we calculated the gradient change (m) of the overall pH change over the CO_2_ range of interest for different base concentrations, where m = ΔpH/Δ%CO_2_ (Figure 5C, green squares). The results clearly show that adding small amounts of NaOH to the system will increase the gradient from 0.5 (<1e-5 M base concentration) to 1 (>0.001 M base concentration), and therefore better sensitivity. We also measured the pH change in the presence of glycerol and noticed the addition of glycerol will significantly lower the pH of the solutions (Figure 5B). Adding glycerol will shift the pH vs. %CO_2_ curves towards the lower pH range in Figure 5A, although the exact mechanism is still unknown. As a result, the pH change gradient for 50% glycerol solutions maximized at 0.05 M NaOH (Figure 5C, red circles), a concentration that is much higher than the theoretical prediction in the absence of glycerol (Figure 5C, green squares). The pH gradient data matched well with the fluorescence measurement data (Figure 4B), which also peaked at around 0.05 NaOH. Beyond this concentration, sensor responsiveness is suppressed at strongly alkaline conditions due to the elevated overall pH condition above the pKa of HPTS (7.7) (Bhosale et al., 2017). Although the pH gradient (m) is maximized at high NaOH concentrations (>0.05 M), the solution is too basic for HPTS to convert from deprotonated forms to protonated forms, and therefore reduces the fluorescence signal fold change.

### Analytical performance

**FIGURE 6:**
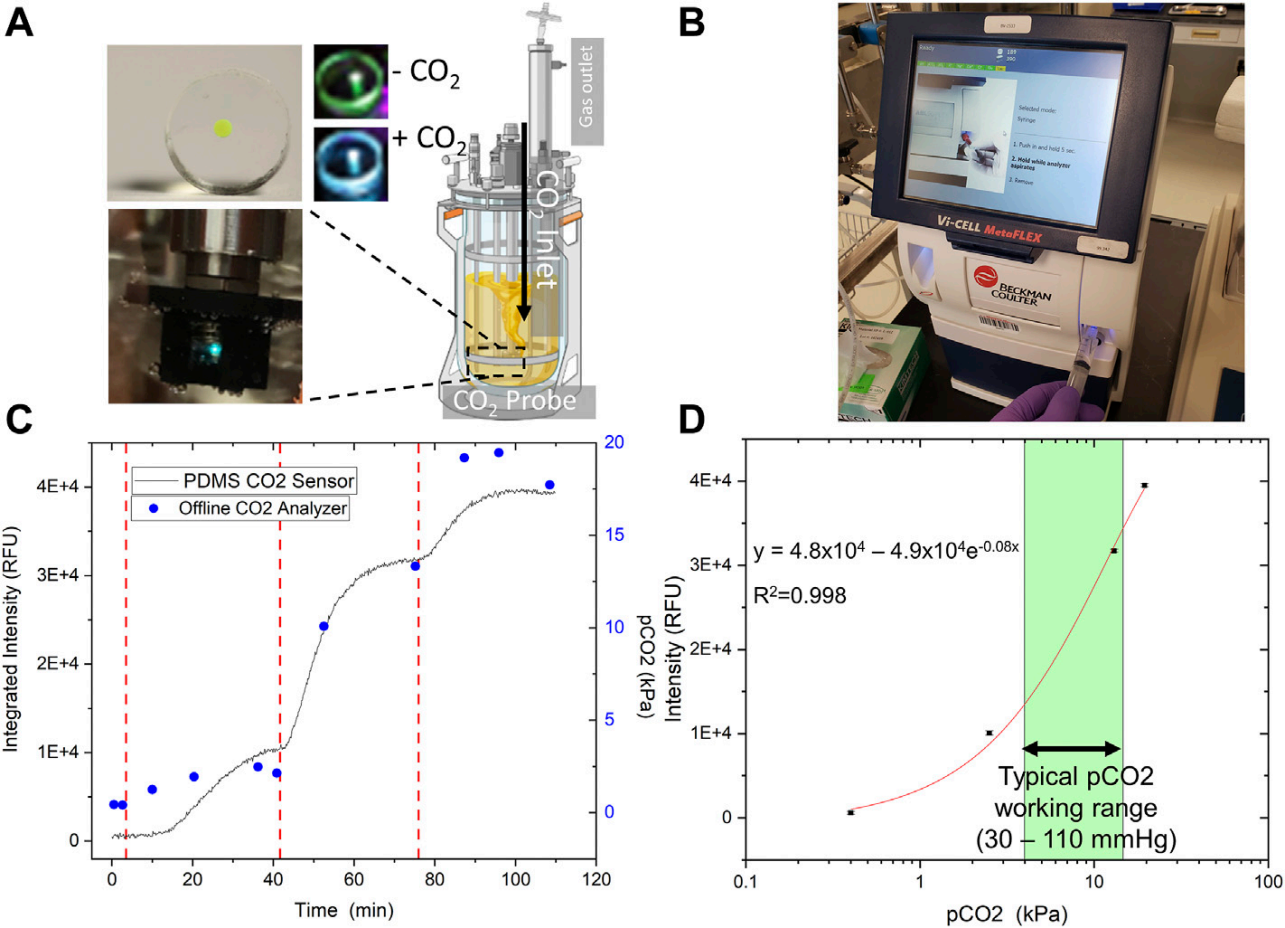
Sensor Analytical Performance. The optimized sensor was tested in-line in a 2 L Sartorius Biostat Bioreactor System **(A)** with incrementally increased pCO_2_, orthogonally measured offline by a Beckman Coulter bioanalyte analyzer **(B)**. **(C)** The trend in the integrated intensity (436–455 nm) is highly similar to offline measurements. The gas flow mix was modulated via bioreactor control tower rotameters to increase CO_2_ partial pressure after equilibration (vertical dashed red lines). **(D)** The sensor was tested up to 20 kPa CO_2_, a typical maximum allowable cell culture concentration, and a non-linear calibration curve was generated. A calibration curve was built for the optimized sensor in two different experiments. The data shown here is representative of both. Our sensor has a strong resolution (< 0.5 kPa), and a working range extending from 0 to at least 20 kPa CO_2_.

We next tested the fabricated CO_2_ sensor to determine its analytical attributes as well as to create a calibration curve for real-time CO_2_ detection. Developing a reliable gas sensor requires the ability to generate a consistent standard curve to interpolate later measurements of unknown concentrations. A 2 L Sartorius Stedim Biostat® B+ cell culture bioreactor served as the testbed for our experiments (Figure 6A), in which various CO_2_ feed rates balanced by nitrogen (N_2_) were sparged into the reactor to vary the partial pressure of CO_2_ dissolved in the water (up to 20 kPa).

CO_2_ was measured in real-time with the fabricated sensor and an offline blood-gas analyzer (Figure 6B) to confirm working pCO_2_ concentration. Overall, the CO_2_ sensor data matched well with the results from the benchtop gas analyzer (Figure 6C). The data was then fitted non-linearly using an exponential fitting function to generate a calibration curve (Figure 6D). If we consider a fairly broad range (Blombach and Takors, 2015) of operating pCO_2_ concentrations from 30—110 mm Hg (or 4—14.7 kPa) to support mammalian cell culture, the CO_2_ sensor provides adequate sensitivity and resolution at the upper end of this range (Figure 6D).

We calculated sensor resolution at each point with a conservative approach using three times the standard deviation of each calibration point (Eq. 7). Adding and subtracting 1.5σ (1.5*standard deviation) from the sensor intensity value at a calibration point ( $f\left(x_{i}\right)$ ), yields an intensity difference of 3σ , a statistically significant difference capable of being reliably resolved. Dividing this value by $f^{\prime }\left(x_{i}\right)$ , the slope of the regression curve at each calibration point returns the difference of pCO_2_ concentrations (*x*-axis values) determined by 3σ . We calculated the resolution (or sensitivity) of our sensor in the operating pCO_2_ range of 30–110 mm Hg. The maximum of x-value differences from the calibration data set within the operating pCO_2_ range is considered the calculated resolution. By doing this, we estimated the analytical resolution of our sensor to be ∼0.5 kPa (3.7 mm Hg, or 0.5% CO_2_). In addition the fabricated CO_2_ sensor also demonstrated a high signal-to-noise ratio, which aids in attaining a high resolution. After optimizing the dye loading chemistry, the sensor’s working range could extend from 0 to beyond 20 kPa, although we did not test higher concentrations to confirm the outer bound.

$$\mathrm{Analytical}\;\mathrm{Resolution}=\frac{3\sigma_{i}}{f^{\prime }\left(x_{i}\right)}$$ (7)

Where $x_{i}=\mathrm{pC}O_{2}$ value of different calibration points;

$\sigma_{i}$ = standard deviation of measurement at calibration point, i ;

$f^{\prime }\left(x_{i}\right)$ = slope of regression curve f , at calibration point, i ;

The response time of the PDMS-based CO_2_ sensor is dependent on a variety of factors. From a sensor design perspective, the thickness and pore size of the PDMS matrix containing HPTS are critical. Specifically, the time to respond to a change in bulk pCO_2_ depends on diffusion through the PDMS. With a total volume of 0.75 μl of dye loaded into the PDMS, we achieved sensor membranes just over 1 mm thick (Figure 3B). A bioreactor may present an ideal scenario to minimize response time with constant fluid mixing. We noticed the sensor response time varied depending upon the rate of increase of CO_2_ in the sparge gas; however, the average response time was about 10 min (Figure 6C). For cell culture monitoring, a 10 min response time is virtually real-time considering industrial offline CO_2_ measurement is typically infrequent. However, a 10 min response time might not be ideal for other applications. Response time can be improved by lowering droplet volume and reducing the PDMS patch thickness.

The analytical attributes of our sensor make it a very attractive option for CO_2_ monitoring in comparison against other relevant sensors as summarized in Table 1. Several other sensors utilizing HPTS can successfully quantify CO_2_ up to 30%. However due to a lower signal-to-noise ratio, many existing CO_2_ sensors have relatively lower resolutions. When considering the range of interest for cell culture monitoring (∼4–14.7%), our fabricated sensor’s resolution is as good or better than commercial options for a fraction of the cost.

**TABLE 1:** Comparison of recent CO_2_ sensor fabrications.

Sensing material	Matrix	Principal	Working range	Resolution	Ref
MCP, SR101	Silicon rubber patch	FRET	0–10%	0.1%	Sipior et al. (1995)
HPTS	Chemically modified PDMS patch	Fluorescence	0–20%	6%	Ge et al. (2005)
HPTS	ORMOSIL sol-gel film	Fluorescence	0.03–30%	4.4%	Dansby-sparks et al. (2010)
HPTS	ORMOSIL sol-gel film	Fluorescence	0–20%	2.8%	MacCraith, (1998)
Mid infrared Sensor (commercial option)	Stainless steel, silicone membrane	NDIR	0.5–100%	0.5%	(Hamilton)
Severinghaus Electrode (commercial option)	Stainless steel, PTFE membrane	Potentiometric Severinghaus	0.1–100%	1%	(Toledo)
HPTS	PDMS patch	Fluorescence	0–20%	0.5%	This work

### Sensor robustness

Analytical sensors can commonly be limited by a range of factors, including limited shelf life, sensitivity to non-target analytes yielding erroneous signals, and degradation at specific environmental conditions, such as temperature, pH, etc. The PDMS-based CO_2_ sensor is resistant to many of these drawbacks due to its design and the robust characteristics of PDMS. PDMS is a remarkably resilient polymeric matrix. In addition to its ability to withstand temperatures of up to 200°C (Sylgard 184 product sheet), it can undergo thousands of bending and stretching cycles without degrading its physical attributes (Chen et al., 2018). The flexibility of PDMS as a stretchable sensor (Figure 3D) makes it a great candidate for wearable sensors for plant and human health monitoring, given the dynamic strain and movement requirements (Chen et al., 2018; Trung and Lee, 2016; Lee et al., 2021). Given the dye sensor is contained within the PDMS hydrophobic polymer matrix and signal change is pH dependent, potential sources of cross-analyte interference are highly limited to species such as acidic vapors that can diffuse into the PDMS, altering pH of the droplet within. In a cell culture environment, acidic species such as lactic acid are present, however we expect the sensor to maintain specificity to CO_2_ given the negligible fraction of lactic acid present in the vapor phase.

**FIGURE 7:**
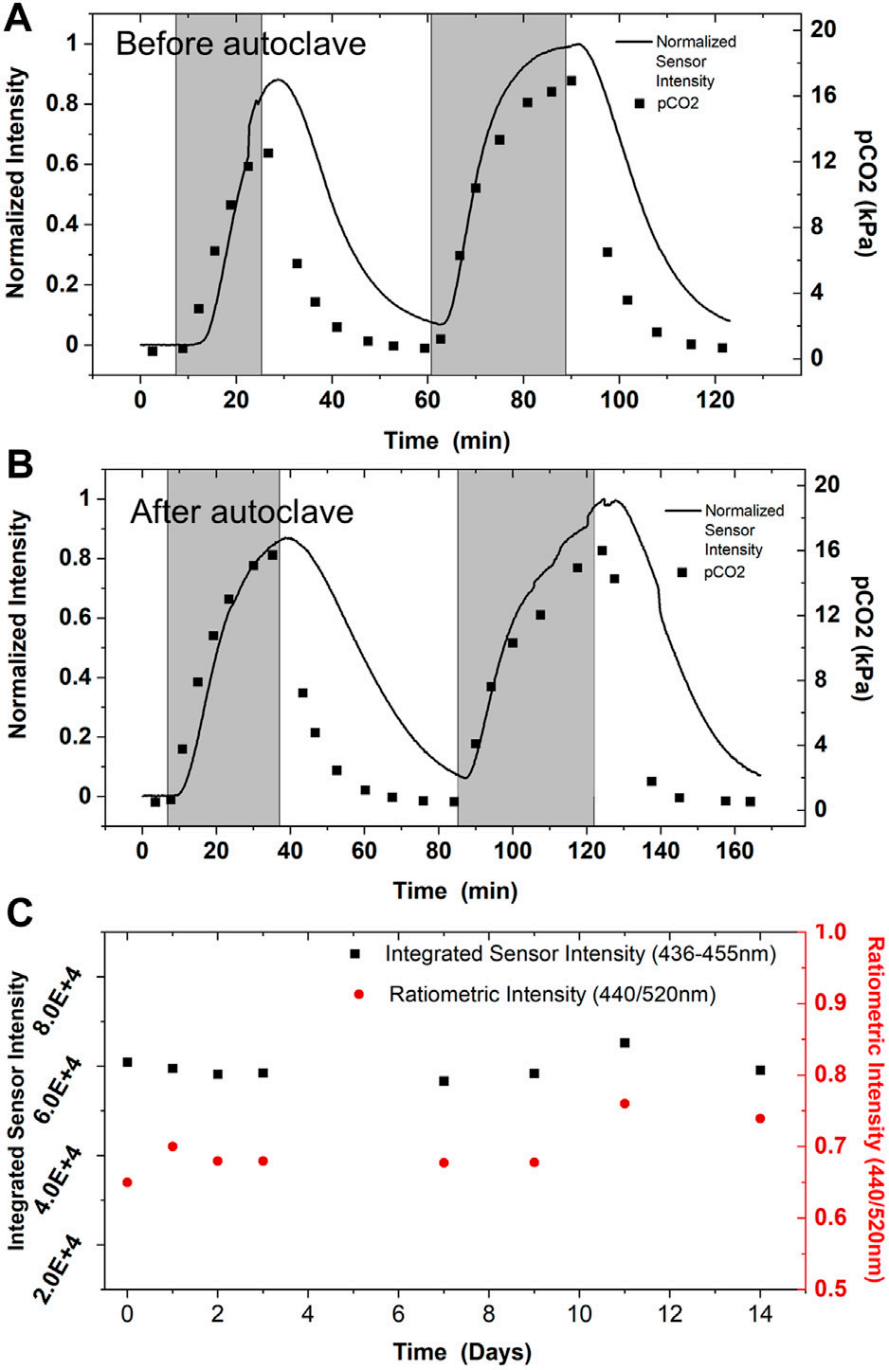
Sensor Robustness. CO_2_ sensor performance in 2 L bioreactor was validated against an offline CO_2_ analyzer prior to autoclave sterilization **(A)**, and after autoclave sterilization **(B)**. Sensor performance was monitored while sparging up to 20 kPa CO_2_ into the reactor (temporally represented by shaded gray region), then CO_2_ levels were allowed to return to baseline levels before repeating. Performance before and after autoclaving is nominally indistinguishable; performance does not appear to be altered by steam sterilization, as expected from the robust physical properties PDMS. Multiple sensors were tested before and after autoclaving without alterations in sensing performance; data shown is representative of multiple experiments. **(C)** The CO_2_ sensor is also shelf stable for long periods of time. Sensor spectra was monitored after exposure to 100% CO_2_ beginning the day of fabrication with minimal shift in performance observed.

In cell culture applications, all bioreactor components must be sterilized. Sterilization is most commonly accomplished through autoclaving for glass vessels, steam-in-place (SIP) for stainless bioreactors, and gamma irradiation for single-use bioreactors. While we did not investigate stability of the sensor after gamma irradiation, the CO_2_ sensor was tested before and after steam sterilization via autoclaving to demonstrate robustness and breadth of potential applications (Figure 7A,B). CO_2_ was sparged into a bioreactor to a total partial pressure of about 20 kPa. Then CO_2_ was removed from the gas mix, and this cycle was repeated. The sensor performance displays a high level of similarity between the tests and agrees with the offline analyzer’s trend (Figures 7A,B). This data and the sensor components’ autoclave compatibility establish the CO_2_ sensor as compatible with autoclave sterilization before deployment in environments requiring sterility.

The CO_2_ sensor is remarkably stable for long periods at room temperature. After fabrication of the PDMS-based sensor, the sensor’s sensitivity was monitored for 2 weeks for any signal degradation or alteration in performance. The sensor was flooded with 100% CO_2_ in the gas-phase, and the signal corresponding to protonated dye was allowed to equilibrate to a stable maximum intensity (Figure 7C). The same sensor was used for data collection over the 2 week period, highlighting its ability for baseline recovery and multiple uses. Overall change in signal was minimal over the experimental time course, indicating the sensor is still fully functional for multiple weeks.

## Conclusion

CO_2_ monitoring has been a focus of analytical device development for almost 70 years. Many accurate devices and sensors have been created and successfully integrated into various fields where CO_2_ quantification is of interest. We developed what we believe is the simplest to fabricate CO_2_ sensor to date that is also capable of continuous monitoring in real-time and is robust enough for long-term stable storage as well as exposure to harsh conditions such as autoclaving. Coupling gravity-based deposition in PDMS with HPTS, we developed a quick sensor fabrication method that is amenable to calibration and subsequent gas quantification. The prepared CO_2_ sensor is able to detect pCO_2_ accurately in a working range of 0–20 kPa with a resolution of 0.5 kPa. Due to the elastic modulus of PDMS, the sensor is capable of bending and stretching, increasing its versatility as an easy-to-use sensor. This highly adaptable format is a powerful method for not only carbon dioxide sensing, but also gas sensing more broadly. In addition to use as a CO_2_ sensor for process monitoring, we envision use of this sensor design as a flexible, stretchable wearable gas sensor for plant and human health monitoring.

## Data Availability

The original contributions presented in the study are included in the article/Supplementary Material; further inquiry can be directed to the corresponding author.

## References

[B1] Bhosale R. S. Shitre G. V. Kumar R. Biradar D. O. Narayan R. (2017). A 8-hydroxypyrene-1, 3, 6-trisulfonic acid trisodium salt (HPTS) based colorimetric and green turn-on fluorescent sensor for the detection of arginine and lysine in aqueous solution. Sensors Actuators B Chem. 241, 1270–1275. 10.1016/j.snb.2016.10.002

[B2] Bielser J. M. Wolf M. Souquet J. Broly H. Morbidelli M. (2018). Perfusion mammalian cell culture for recombinant protein manufacturing – a critical review. Biotechnol. Adv. 36 (4), 1328–1340. 10.1016/j.biotechadv.2018.04.011 29738813

[B3] Blombach B. Takors R. (2015). CO2 - intrinsic product, essential substrate, and regulatory trigger of microbial and mammalian production processes. Front. Bioeng. Biotechnol. 3 (AUG), 108–111. 10.3389/fbioe.2015.00108 26284242 PMC4522908

[B4] Bort J. A. H. Hackl M. Hoflmayer H. Jadhav V. Harreither E. Kumar N. (2012). Dynamic mRNA and miRNA profiling of CHO-K1 suspension cell cultures. Biotechnol. J. 7 (4), 500–515. 10.1002/biot.201100143 21751394

[B5] Chatterjee C. Ayusman S. (2015). Sensitive colorimetric sensors for visual detection of carbon dioxide and sulfur dioxide. J. Mat. Chem. A 3, 5642–5647. 10.1039/c4ta06321j

[B6] Chen J. Zheng J. Gao Q. Zhang J. Zhang J. Omisore O. (2018). Polydimethylsiloxane ( PDMS ) -based flexible resistive strain sensors for wearable applications. Appl. Sci. (Basel). 8 (3), 345. 10.3390/app8030345

[B7] Chu C. Lo Y. (2008). Fiber-optic carbon dioxide sensor based on fluorinated xerogels doped with HPTS. Sensors Actuators B Chem. 129, 120–125. 10.1016/j.snb.2007.07.082

[B8] Chu C. Lo Y. (2009). Highly sensitive and linear optical fiber carbon dioxide sensor based on sol – gel matrix doped with silica particles and HPTS. Sensors Actuators B Chem. 143, 205–210. 10.1016/j.snb.2009.09.019

[B9] Dansby-sparks R. N. Jin J. Mechery S. J. Sampathkumaran U. Owen T. W. Yu B. D. (2010). Fluorescent-dye-Doped sol - gel sensor for highly sensitive carbon dioxide gas detection below atmospheric concentrations. Anal. Chem. 82 (2), 593–600. 10.1021/ac901890r 20038093

[B10] Food and Drug Administration, “Guidance for industry, PAT-A framework for innovative pharmaceutical development, manufacturing and quality assurance,” 2004, [Online]. Available at: http://www.fda.gov/downloads/Drugs/GuidanceComplianceRegulatoryInformation/Guidances/ucm070305.pdf (Accessed April 27, 2022).

[B11] Faccio G. Cont A. Mailand E. Malini R. I. Maniura K. Rossi R. M.,“Complete inclusion of bioactive molecules and particles in polydimethylsiloxane : A straightforward process under mild conditions,” Scientific Reports 9, pp. 1–8. 2019, 10.1038/s41598-019-54155-5 31772250 PMC6879495

[B12] Ge X. Kostov Y. Rao G. (2005). Low-cost noninvasive optical CO2 sensing system for fermentation and cell culture. Biotechnol. Bioeng. 89 (3), 329–334. 10.1002/bit.20337 15625676

[B13] Hamilton, “Co2ntrol specification shet.” .

[B14] Lee G. Wei Q. Zhu Y. (2021). Emerging wearable sensors for plant health monitoring. Adv. Funct. Mat. 31 (52), 2106475–2106514. 10.1002/adfm.202106475

[B15] MacCraith B. D. (1998). Dye-doped organically modified silica glass for fluorescence based carbon dioxide gas detection. Analyst 123 (11), 2373–2376. 10.1039/a805803b

[B16] Malins C. Maccraith B. D. (1998). Dye-doped organically modified silica glass for fluorescence based carbon dioxide gas detection. Analyst 123, 2373–2376. 10.1039/a805803b

[B17] Mills A. Chang Q. (1994). Colorimetric polymer film sensors for dissolved carbon dioxide. Sensors Actuators B Chem. 21 (2), 83–89. 10.1016/0925-4005(94)80008-1

[B18] Mills A. Chang Q. (1993). Fluorescence plastic thin-film sensor for carbon dioxide. Analyst 118, 839–843.

[B19] Mills A. Chang Q. (1994). Tuning colourimteric and fluorimetric gas sensors for carbon dioxide. Anal. Chim. Acta X. 285, 113–123. 10.1016/0003-2670(94)85015-1

[B20] Mills A. (2009). “Optical sensors for carbon dioxide and their applications,” in Sensors for environment, health, and safety, 347–370.

[B21] Neethirajan S. Jayas D. S. Sadistap S. (2009). Carbon dioxide (CO2) sensors for the agri-food industry-A review. Food bioproc. Tech. 2 (2), 115–121. 10.1007/s11947-008-0154-y

[B22] Park J. B. K. Craggs R. J. (2010). Wastewater treatment and algal production in high rate algal ponds with carbon dioxide addition. Water Sci. Technol. 61 (3), 633–639. 10.2166/wst.2010.951 20150699

[B23] Rao H. X. Liu F. N. Zhang Z. Y. (2007). Preparation and oxygen/nitrogen permeability of PDMS crosslinked membrane and PDMS/tetraethoxysilicone hybrid membrane. J. Memb. Sci. 303 (1–2), 132–139. 10.1016/j.memsci.2007.07.002

[B24] Severinghaus J. W. Bradley A. F. (1958). Electrodes for blood pO2 and pCO2 determination. J. Appl. Physiol. 13 (3), 515–520. 10.1152/jappl.1958.13.3.515 13587443

[B25] Sipior J. Bambot S. Romauld M. Carter G. M. Lakowicz J. R. Rao G. (1995). A lifetime-based optical CO2 gas sensor with blue or red excitation and Stokes or anti-Stokes detection. Anal. Biochem. 227 (2), 309–318. 10.1006/abio.1995.1286 7573952 PMC6911361

[B26] Toledo M. CO2 sensor InPro 5000i technical data sheet.

[B27] Trung T. Q. Lee N. (2016). Flexible and stretchable physical sensor integrated platforms for wearable human-activity monitoring and personal healthcare. Adv. Mat. 28 (22), 4338–4372. 10.1002/adma.201504244 26840387

[B28] Wolfbeis O. S. Kovács B. Goswami K. Klainer S. M. (1998). Fiber-optic fluorescence carbon dioxide sensor for environmental monitoring. Mikrochim. Acta 129 (3), 181–188. 10.1007/bf01244739

