# Supplementary material for: Flexible sensor patch for continuous carbon dioxide monitoring
Source: Front Chem. 2022 Sep 27;10:983523. doi: 10.3389/fchem.2022.983523 (PMC9552331; doi:10.3389/fchem.2022.983523)
Supplement: Supplementary file 2 [file DataSheet1.docx]

**Supplementary Materials**

Flexible Sensor Patch for Continuous Carbon Dioxide Monitoring

Zach Hetzler^1^, Yan Wang^1^, Danny Krafft^2^, Sina Jamalzadegan^1^, Laurie Overton^3^, Michael Kudenov^2^, Frances Ligler^4^, Qingshan Wei^1*^

^1^Department of Chemical and Biomolecular Engineering, NC State University, Raleigh, NC, USA

^2^Department of Electrical and Computer Engineering, NC State University, Raleigh, NC, USA

^3^Biomanufacturing Training and Education Center (BTEC), NC State University, Raleigh, NC, USA

^4^Department of Biomedical Engineering, Texas A&M University, College Station, TX, USA

*** Correspondence:** Qingshan Wei (qwei3@ncsu.edu)


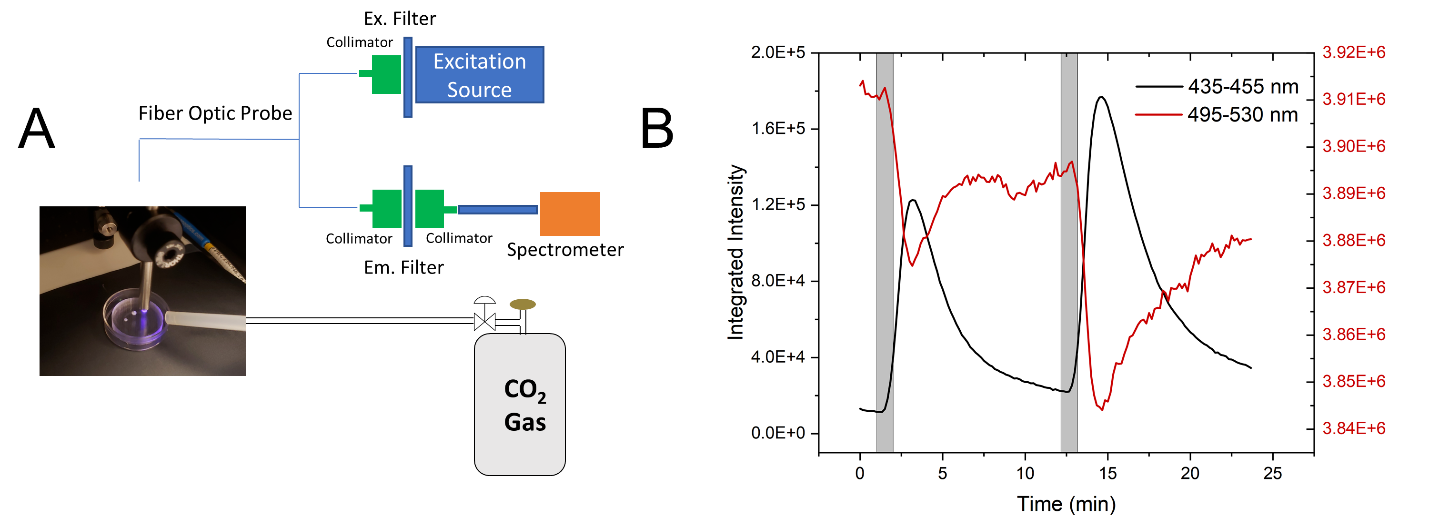


**Figure S1. Preliminary evaluation of sensor performance**. (A) Multiple sensor formats were initially tested in gas-phase to screen for sensor responsiveness. Briefly, CO_2_ gas was flowed over the sensor while monitoring with our benchtop optical set-up while exciting at 405 nm and monitoring spectral bands corresponding to the protonated dye (red curve) and deprotonated dye (black curve). (B) We found that excitation at 405 nm provided a more significant response from the protonated dye spectra (435-455 nm).


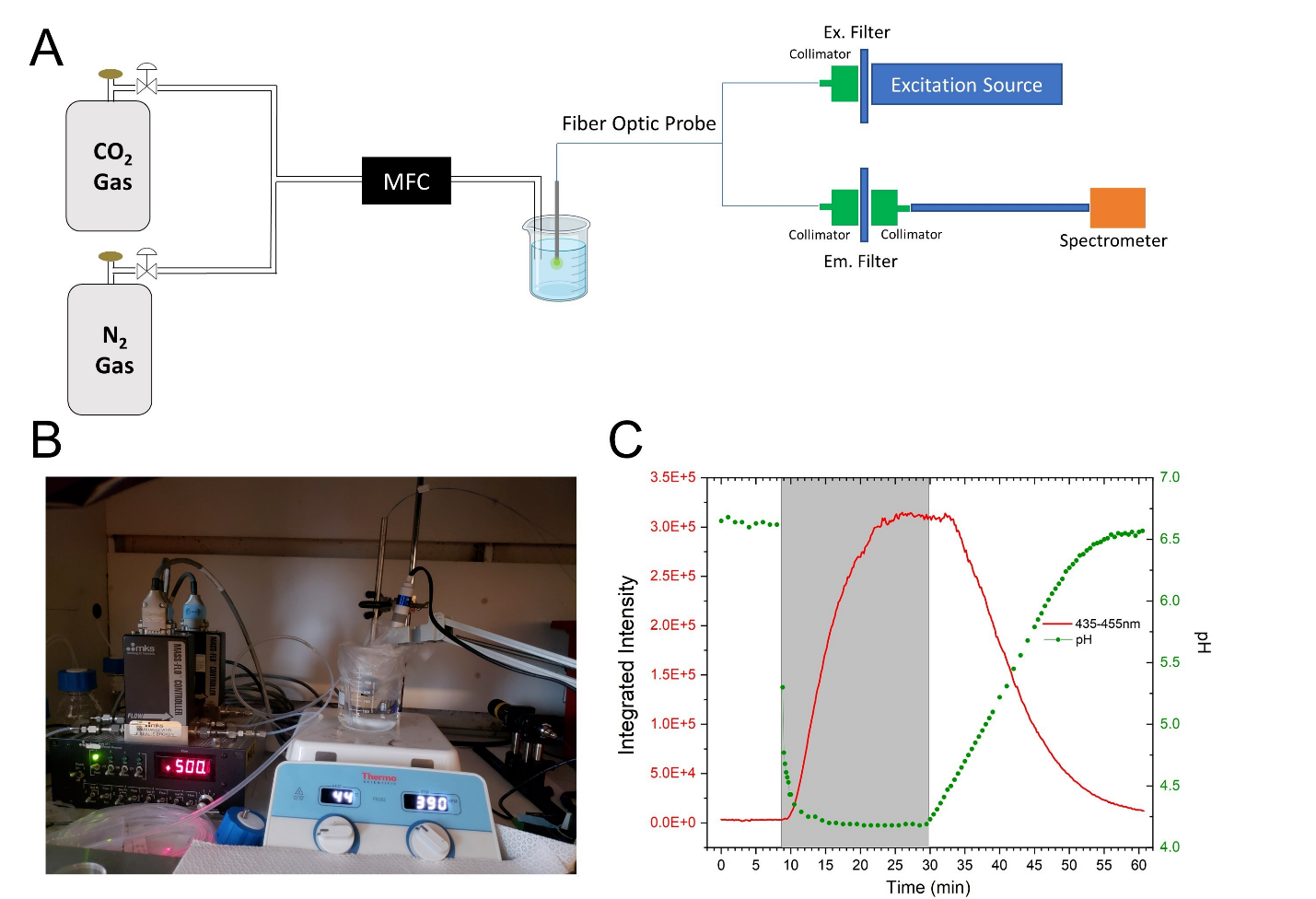


**Figure S2. Lab-scale liquid phase sensor demonstration**. (A) We next challenged the CO_2_ sensor in the liquid-phase by bubbling CO_2_ into water through a mass flow controller (MFC). (B) A beaker with water was placed on a hot plate to achieve 37ºC while bubbling 50% CO­_2_ into the liquid (gray area) while monitoring pH with a benchtop pH meter as well as sensor response. (C) Response of the protonated dye measured at 435-455 nm as well as pH; the signals from the dye and pH are inversely proportional, as expected based on the kinetics of CO_2_ reaction with water and fluorescent properties of HPTS.


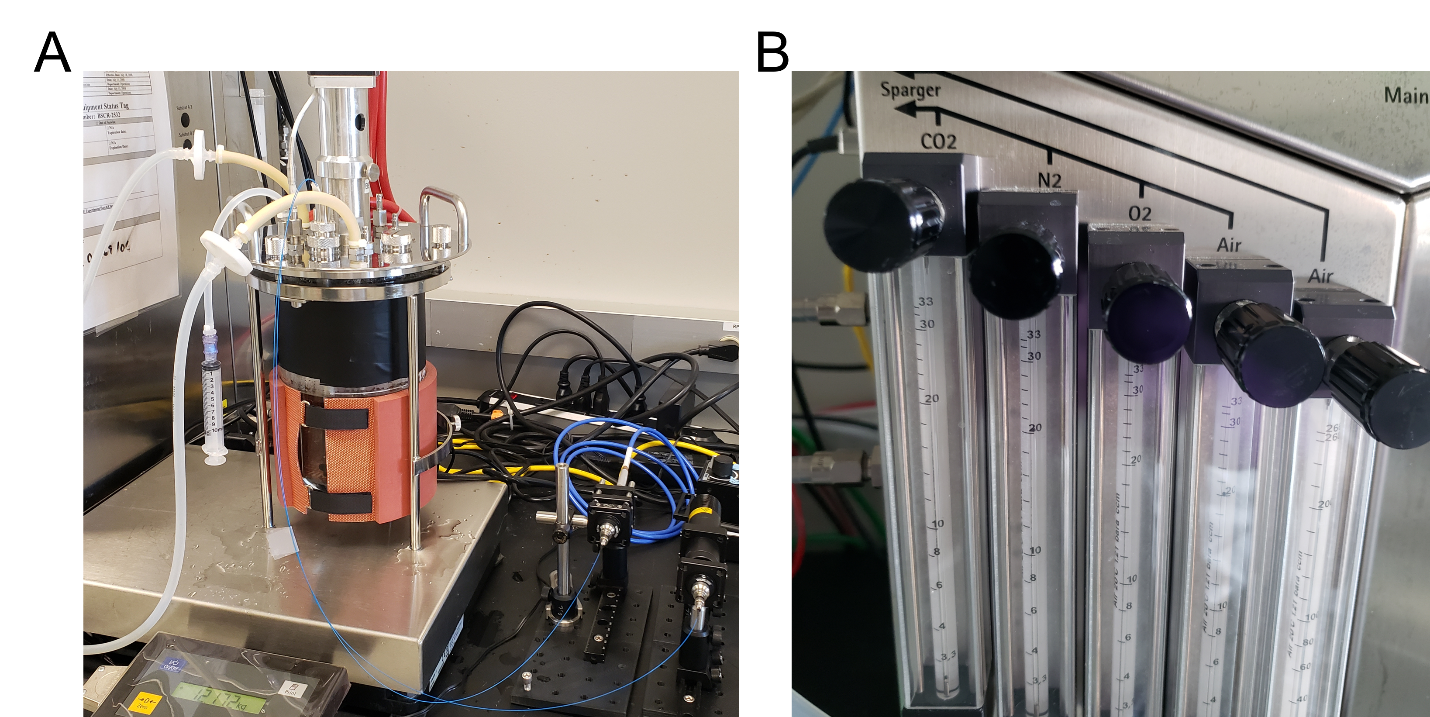


**Figure S3. 2L Bioreactor CO_2_ testing set-up.** (A) The CO_2_ sensor was tested in a real fermentation environment in a 2L Sartorius Stedim Biostat B plus bioreactor. CO_2_, balanced by N_2_, was delivered through the headplate and sparged into the reactor via a circular sparge ring. Temperature was maintained at 37ºC, and liquid was withdrawn via syringe for offline CO_2_ measurements. (B) CO_2_ partial pressure of the sparging gas was managed by rotameter control. The bioreactor was vented, thus percent composition of gas flow determined partial pressure.


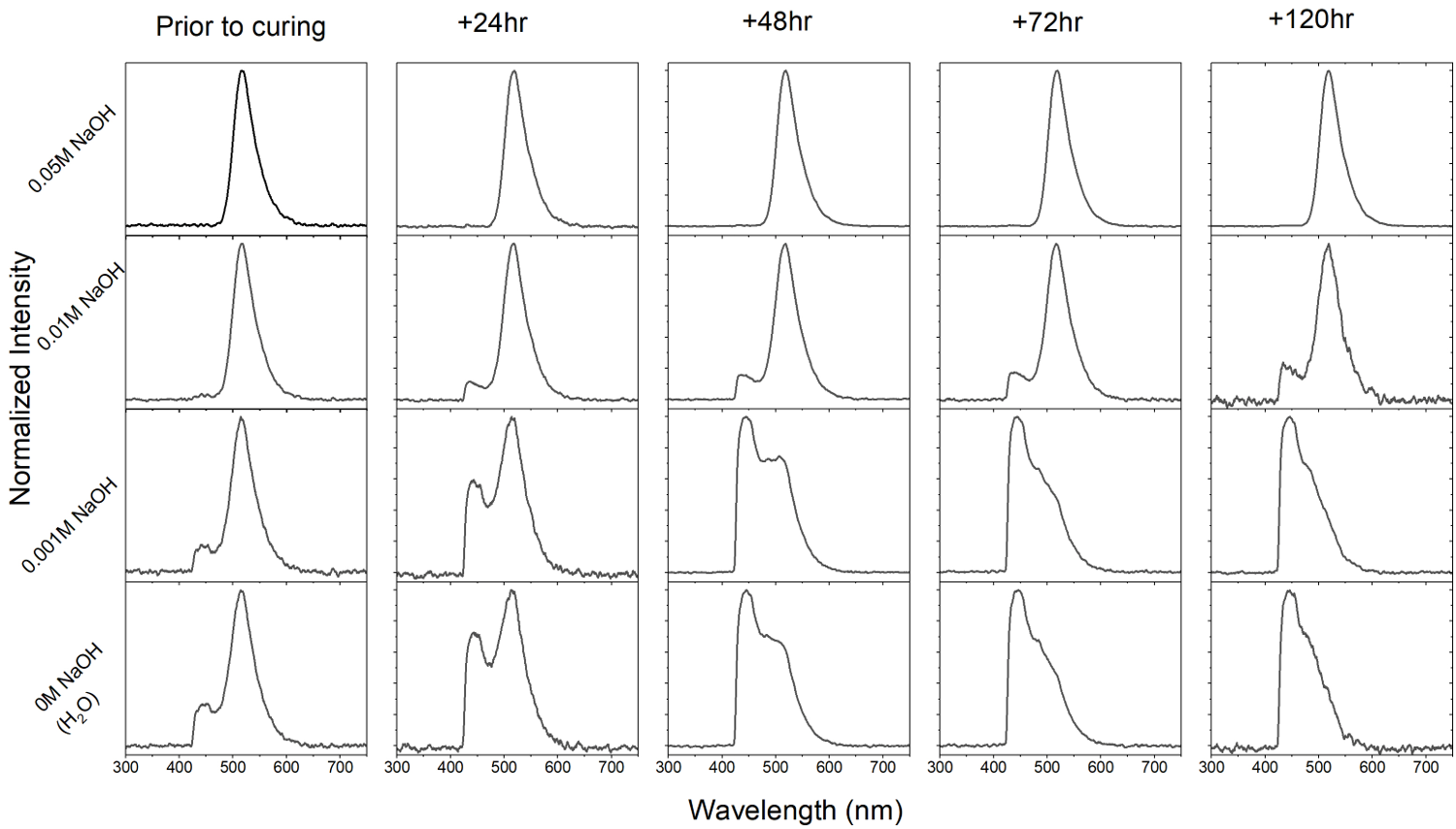


**Figure S4. Effect of base concentration on baseline fluorescent spectra.** After preparation of several sensors, we noticed alterations in baseline fluorescent signal prior to exposure to CO_2_ in an experimental setting. To investigate the role of base concentration on sensor stability, we evaluated fluorescent spectra of HPTS before curing and for several days afterwards. The PDMS-based CO_2_ sensors with ≤0.05 M NaOH displayed an increase in the spectral peak corresponding to the protonated dye (~450 nm) in as little as 24 hours after fabrication. There is also a clear correlation of increased ratio of protonated dye with decreasing concentration of NaOH. With lower NaOH concentration, the area under the protonated dye peak centered around 450 nm increases. Increased protonated peak area is indicative of dye acidification, likely by CO_2_. This suggests this simple sensor format can easily detect the carbon dioxide in the atmosphere (0.04%), and likely even lower concentrations.


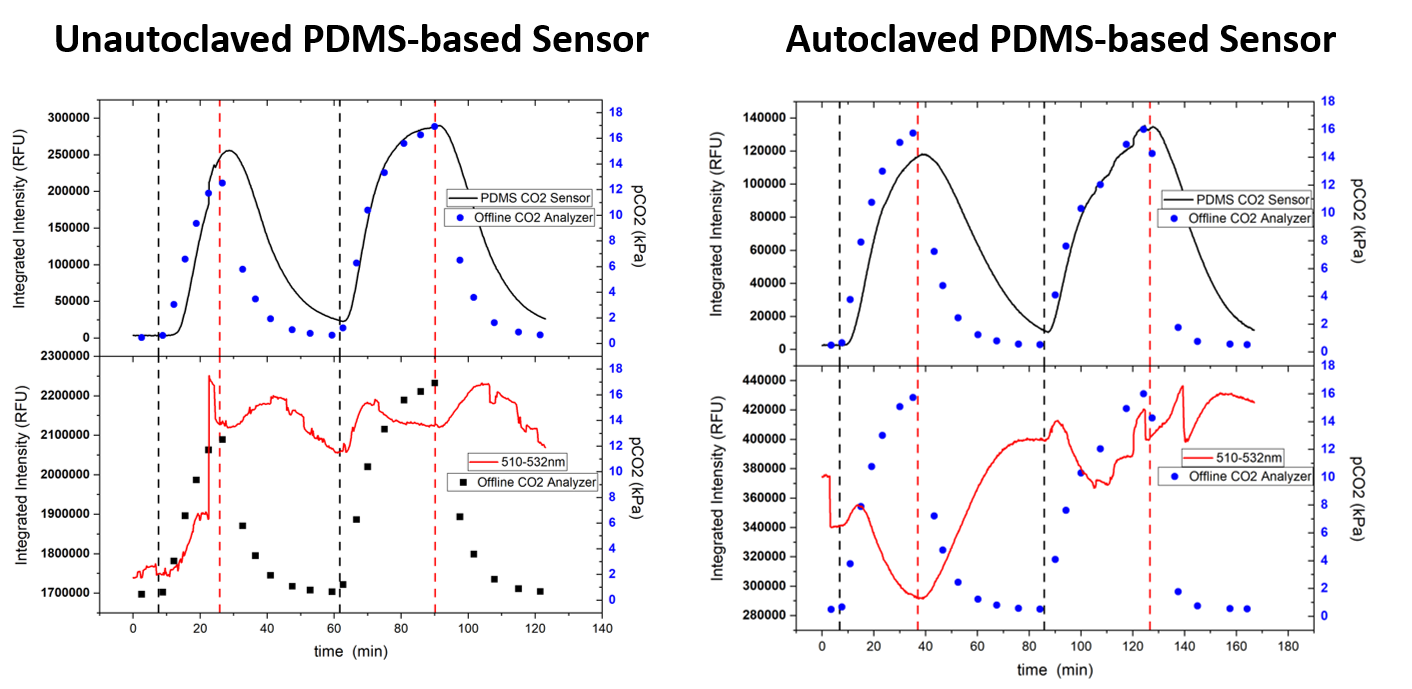


**Figure S5. Sensor response channel integrity after autoclaving**. The CO_2_ sensor was monitored using two detection channels corresponding to the protonated and deprotonated forms of the dye for both before and after autoclaving. Exciting the dye at 405 nm returned very reliable signal responses from the protonated dye channel (black curves), and less responsive and erratic data from the deprotonated channel (red curves).

**Supporting Video S1: Preparation steps of the thin-film CO_2_ sensor patch**
